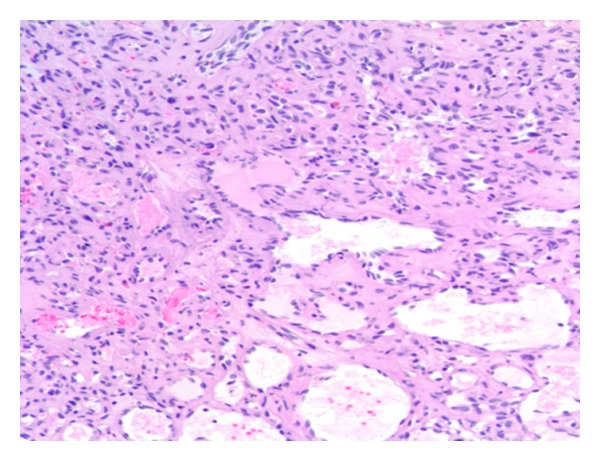# Lobular Capillary Hemangioma in the Posterior Trachea: A Rare Cause of Hemoptysis

**DOI:** 10.1155/2012/592524

**Published:** 2012-11-27

**Authors:** Ford Turner Amy, Diaz-Guzman Enrique

**Affiliations:** Division of Pulmonary, Critical Care and Sleep Medicine, Department of Internal Medicine, Chandler Hospital and Kentucky Clinic, University of Kentucky, Lexington, KY 40536, USA

## Abstract

Lobular capillary hemangiomas (LCH) have been cited in the literature as a rare potential cause for recurrent hemoptysis. They are mostly associated with cutaneous and mucosal surfaces. Rarely do they affect the trachea and associated airways in adults. Lobular capillary hemangiomas can be associated with previous trauma to the airway; however, drugs, hormonal shifts, viral oncogenes, production of angiogenic factors, and cytogenetic clonal deletion abnormalities can also influence these lesions. We document a case of a posterior wall tracheal hemangioma with associated recurrent hemoptysis in a 22-year-old male on testosterone therapy. An LCH attached to the posterior tracheal wall can be technically difficult to remove due to risk of perforation and bleeding. There have been no cases documented of posterior wall LCH.

## 1. Introduction 

Lobular capillary hemangiomas (LCH), formally known as pyogenic granuloma, usually present as a painless, bleeding mass adherent to the mucosal or cutaneous surfaces of the upper airways. Most commonly they are present on the lip, nose, oral cavity, and tongue. According to a pathologic review of 639 vascular lesions of the upper airway [1], they are more common in males less than 18 years and females of reproductive age. Most cases reviewed involve pediatric patients. They have only recently been documented in the trachea [2–6]. No cases have been reported of a posterior wall LCH. We report on a 22-year-old male that presented with recurrent hemoptysis. Upon bronchoscopic evaluation, he had an adherent vascular lesion pathologically found to be a LCH attached to his posterior tracheal wall.

## 2. Case Presentation

 A 22-year-old male presented to our clinic with recurrent episodes of hemoptysis over a 4-week period with 3 distinct events. He stated that this started with an incidence of heavy coughing. He then would cough up to 2–4 tablespoons of blood. After presentation to the emergency room, it was initially thought this was related to epistaxis, and he was sent home with treatment for allergic rhinitis. He had no other symptoms, denying fever, weight loss or gain, chest pain, easy bruising, dyspnea, or rash. He did complain of chronic postnasal drip, nasal congestion, and occasional pruritis, which he attributed to allergies. He had a past medical history of eosinophilic colitis, obsessive-compulsive disorder, history of an aneurismal bone cyst status post-removal, and allergic rhinitis. He had also been recently diagnosed with hypogonadism over the last 5 months, and was on injectable testosterone supplementation. He denied smoking, alcohol, and illicit drug use and had no known inhalant exposures and trauma to the airway that he could recall. His family history included a brother with eosinophilic colitis and his mother had vascular ectasias of her spinal cord. He did note that these episodes seemed to occur 5 days after his dose of intramuscular testosterone. Physical exam was otherwise unremarkable and laboratory values were unrevealing. Computed tomography did not reveal any notable lesion of the upper airway or lung parenchyma. 

**Figure 1:**
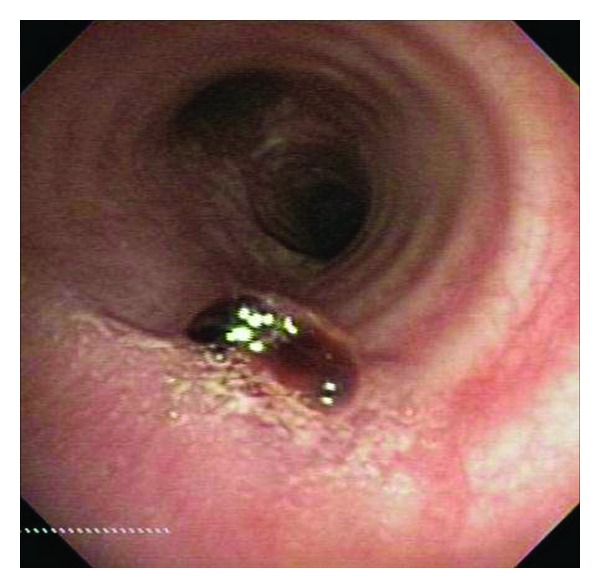


**Figure 2:**
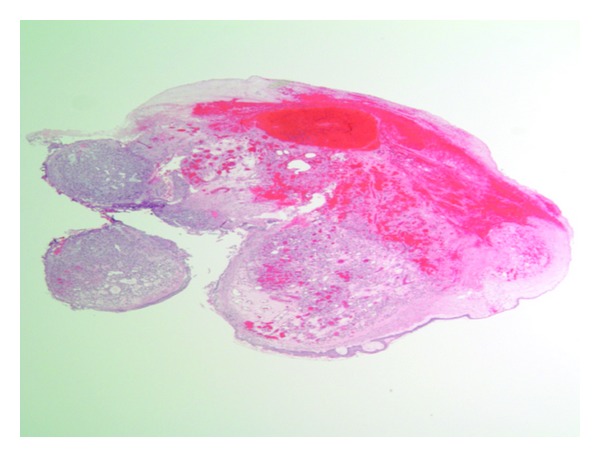


 Upon flexible bronchoscopic evaluation, the following lesion was seen in the distal trachea (Figure 1). The purple, vascular lesion was approximately 1–1.5 cm in size located 3 cm from the carina at the 5 o'clock position along the posterior wall attached by a short pedicle. A biopsy was taken at that time which only showed fibrin products and inflammatory cells. Hemostasis was difficult to obtain without use of cold saline, several injections of topical epinephrine and Argon Plasma Coagulation. Due to unavailability of pathology and the location of the lesion, the patient was taken to the operating suite where a therapeutic flexible bronchoscope was used with utilized. The lesion was obtained by electrocautery loop snare and endoscopic basket. The pathology revealed the diagnosis of lobular capillary hemangioma (Figures 2 and 3). The superficial portions of the lesion had undergone secondary, nonspecific changes including stromal edema, capillary dilation, inflammation, and a granulation tissue reaction. It demonstrated diagnostic, lobular arrangements of capillaries at its base, which consisted of discrete clusters of endothelial cells. The cellular architecture was made up of an inflammatory infiltrate of neutrophils and monocytes.

 Upon followup, the patient had no hemoptysis episodes and has subsequently stopped his testosterone therapy.

## 3. Discussion

 Lobular capillary hemangioma is a well-known entity that commonly involves skin and oral/nasal mucosa, developing rapidly over a number of days to weeks. Its cause is not well defined but certain postulates correlate to previous trauma, hormonal shifts, viral oncogenes, Bartonella infection, production of angiogenic factors, and cytogenetic clonal deletion abnormalities, although certain studies have disproven some of the above [7, 8]. Other causal relationships suggest certain drugs can also cause these lesions, such as retinoid therapy [9] and others. Our patient had recently been started on testosterone therapy, which could be coincidental or have influenced the necessary hormonal shifts to promote such a vascular lesion in a susceptible host. These lesions have commonly and extensively been studied in late pregnancy, arising mostly in the airway mucosa from the influence of female hormones on vascular development [10]. Though the relationship between testosterone and angiogenesis has been correlated in bench studies [11, 12], there has yet to be specific research regarding its role in this particular type of tumor in this instance. Also of note is that our patient had a previous history of a vascular lesion in his tibia that had been removed at the age of 16, requiring surgery and an oral endotracheal intubation. His mother had vascular lesions in her spine found incidentally on imaging, suggesting a genetic proclivity in his family. 

 Regardless of the causal relationships, the position of the lesion became technically difficult due to the posterior wall location and the vascular structure of the tumor itself. The treatment in general for these lesions depends on the extent or size of the lesion, age, comorbidities, and other factors contributing to the overall clinical scenario. Because of inherent thinness of the posterior tracheal wall, there is a risk of iatrogenic tears and perforation. These defects are treated with various approaches; surgical intervention is sometimes required [13]. Lobular capillary hemangiomas have been successfully treated with various other methods involving cryotherapy, YAG laser therapy [14], topical or intralesional steroids or neoplastic agents, propranolol (in children), and surgical excision [15]. If bleeding is a potential risk, such as it is with vascular tumors, having interventional bronchoscopy (e.g., rigid bronchoscopy) available during the removal would be highly suggested. The lesion in this case was easily removed with flexible bronchoscopy, and hemostasis was controlled due to the small pedicle size. Our patient did not experience any complications and is currently symptom free. 

## 4. Conclusion

 Lobular capillary hemangioma found in the trachea continues to be a rare entity and has yet to be described abutting the posterior wall. Posterior laryngeal wall lesions can be challenging due to risk of laceration and perforation and the appropriate equipment needs to be readily available to avoid complications. Though no direct causal link could be found in this patient, we believe that hormonal supplementation may have played a role in its development.

## Figures and Tables

**Figure 3:**